# Lassa and Marburg viruses elicit distinct host transcriptional responses early after infection

**DOI:** 10.1186/1471-2164-15-960

**Published:** 2014-11-06

**Authors:** Ignacio S Caballero, Judy Y Yen, Lisa E Hensley, Anna N Honko, Arthur J Goff, John H Connor

**Affiliations:** Bioinformatics Graduate Program, Boston University, 24 Cummington St, Boston, MA 02215 USA; Department of Microbiology, Boston University School of Medicine, Boston, MA 02118 USA; Virology Division, United States Army Medical Research Institute of Infectious Diseases, Fort Detrick, MD 21702 USA; Integrated Research Facility at Fort Detrick, National Institute of Allergy and Infectious Diseases, National Institutes of Health, Fort Detrick, MD 21702 USA

**Keywords:** Transcriptomics, Transcriptional response, Lassa virus, Marburg virus, Interferon-stimulated genes, Biomarker, Gene expression profile, Early stage diagnostics

## Abstract

**Background:**

Lassa virus and Marburg virus are two causative agents of viral hemorrhagic fever. Their diagnosis is difficult because patients infected with either pathogen present similar nonspecific symptoms early after infection. Current diagnostic tests are based on detecting viral proteins or nucleic acids in the blood, but these cannot be found during the early stages of disease, before the virus starts replicating in the blood. Using the transcriptional response of the host during infection can lead to earlier diagnoses compared to those of traditional methods.

**Results:**

In this study, we use RNA sequencing to obtain a high-resolution view of the *in vivo* transcriptional dynamics of peripheral blood mononuclear cells (PBMCs) throughout both types of infection. We report a subset of host mRNAs, including heat-shock proteins like HSPA1B, immunoglobulins like IGJ, and cell adhesion molecules like SIGLEC1, whose differences in expression are strong enough to distinguish Lassa infection from Marburg infection in non-human primates. We have validated these infection-specific expression differences by using microarrays on a larger set of samples, and by quantifying the expression of individual genes using RT-PCR.

**Conclusions:**

These results suggest that host transcriptional signatures are correlated with specific viral infections, and that they can be used to identify highly pathogenic viruses during the early stages of disease, before standard detection methods become effective.

**Electronic supplementary material:**

The online version of this article (doi:10.1186/1471-2164-15-960) contains supplementary material, which is available to authorized users.

## Background

Lassa fever and Marburg fever are acute viral hemorrhagic illnesses. Lassa Fever is caused by the Lassa virus and it is endemic to a large region of West Africa, where 100,000-300,000 cases have been estimated to occur each year [1]. More than 20 cases have been imported to the United States, Canada, United Kingdom and Japan since 1969, when the disease was first recognized [2, 3]. The case fatality rate in hospitalized patients has recently been reported to be 69% [4]. Marburg fever is a viral hemorrhagic illness caused by the Marburg virus. Several outbreaks have been reported in the Democratic Republic of the Congo [5] and Angola [6] with case fatality rates approaching 90%. Travelers visiting Uganda have also imported the virus into the Netherlands [7] and the United States [8]. Both diseases lack effective therapeutics and early diagnostics.

Early clinical symptoms of infection are shared between different hemorrhagic fevers, and they resemble those of a flu-like illness: fever, headache, myalgia, sore throat, vomiting, diarrhea and dry cough [9]. During the 2014 West Africa Ebola outbreak, samples from suspected patients were routinely tested to rule out infection by Lassa virus, since both pathogens were present in the region and distinguishing between them was an important part of the differential diagnosis [10, 11].

Current diagnostic tests for Lassa and Marburg fevers include enzyme-linked immunosorbent assays (ELISA) and reverse transcriptase polymerase chain reaction (RT-PCR) [12–14]. Because these methods rely on the direct detection of viral proteins or nucleic acids in the blood, they are not useful during the incubation period of the virus, which happens during the early stages of infection [9, 13]. Additionally, most potential therapies for these pathogens—ribavirin [15], post-infection vaccination [16], and stable nucleic acid-lipid particle (SNALP) treatment [17]—work best when administered early. Developing tests that diagnose these infections during the early stages of disease would positively impact survival.

**Figure 1:**
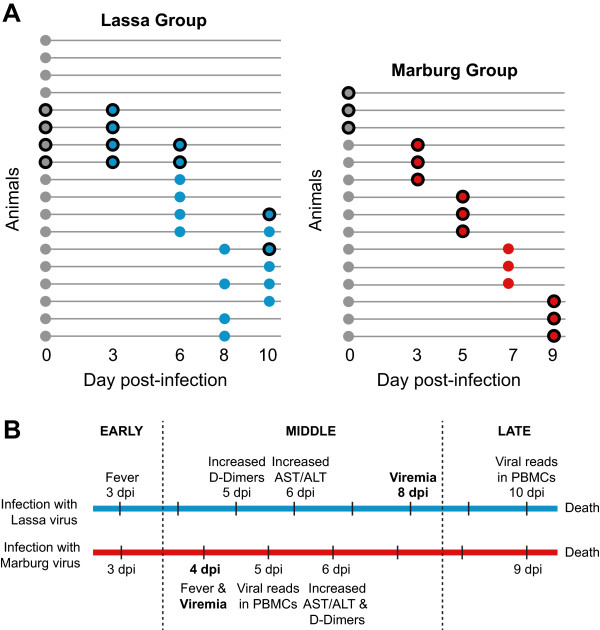
**Quantified samples in each virus group. (A)** Each circle represents an RNA sample isolated from the peripheral blood mononuclear cells (PBMCs) of a cynomolgus macaque. Grey indicates pre-infection samples, each coming from a different cynomolgus macaque. Blue indicates samples infected with Lassa virus, and red indicates samples infected with Marburg virus. All samples were quantified using microarrays. Samples with a black border were also quantified using RNA sequencing. **(B)** Diagram of the disease timecourse highlighting the most relevant clinical events, from the appearance of fever 3 days post-infection to the late stage of disease and death (see also Table 1).

One approach for developing more rapid diagnostics involves incorporating the host response to infection as part of the diagnostic assay. During an infection, circulating immune cells are present in high numbers and they respond to distress signals from many different tissues by altering the levels of expression of specific genes. These changes in expression often take place before the virus starts replicating in the blood (viremia) [18, 19] (Figure 1C), which means that they could be used to perform earlier diagnoses than those allowed by traditional methods. To ensure that the predictive power of these changes in expression can distinguish between different causative agents, the host gene expression profiles associated with each type of infection must be determined in advance. Two recent reports have used a host response signature to discriminate symptomatic patients with viral infections from those infected with bacteria [20, 21]. An RT-PCR assay has also been developed and clinically tested [20].

We have analyzed the transcriptome of PBMCs from non-human primates (cynomolgus macaques) exposed to Lassa virus [18] and to Marburg virus [Lee et al*.*, manuscript in preparation], and we have found that the circulating immune cells develop a robust antiviral response days before clinical symptoms. In this article, we show that the expression differences in the genes that make up this response can be used to distinguish between Lassa and Marburg infections as early as 3 days after exposure.

## Results

### Clinical symptoms

Animals were exposed to an aerosol dose of 1000 plaque forming units of Lassa [18] or Marburg virus [Lee et al., manuscript in preparation], as previously described. In these models, animals began to develop a fever around day 3 after exposure to Lassa virus and day 4 after exposure to Marburg virus (Table 1). The onset of viremia took place around day 8 for Lassa and day 4 for Marburg (confirmed by both plaque assay and RT-PCR). The liver enzymes aspartate transaminase (AST) and alanine transaminase (ALT) showed increased levels of concentration in both diseases around day 6. D-dimers started to increase around day 5 for Lassa and day 6 for Marburg. In both types of infection, all of these markers peaked during the late stages of infection (days 9–10). These data confirmed that clinical markers of infection that could be used for diagnosis (e.g. viremia) did not appear in these models until day 4 post-infection.

**Table 1:** **Times at which clinical markers are detected in each type of infection**

	Lassa	Marburg
Fever	Day 3	Day 4
Viremia	Day 8	Day 4
Increased AST/ALT	Day 6	Day 6
Increased D-dimers	Day 5	Day 6

Viremia was measured using RT-PCR in the Lassa group, and RT-PCR and plaque assay in the Marburg group.

### RNA sequencing

To determine the transcriptional changes that take place in the circulating immune system in response to a hemorrhagic fever virus infection, we quantified the abundance of mRNAs present in peripheral blood mononuclear cells (PBMCs) of cynomolgus macaques infected with either Lassa or Marburg virus. The samples were obtained before the animals were infected, and at multiple times after infection (see Figure 1A). We selected for polyadenylated transcripts and sequenced 12 RNA samples from each group using Illumina technology. This process generated an average of 46.27 ± 5.92 million reads per sample; 72% of which (33.46 ± 4.33 million) aligned to the *M. mulatta* (Rhesus macaque) genome—the closest organism to *M. fascicularis* with an annotated genome. Multiple factors may explain why 28% of reads remained unaligned: an incomplete assembly of the *M. mulatta* genome and transcriptome, genetic differences between *M. mulatta* and *M. fascicularis*, or the appearance of amplification artifacts during the sequencing process. We assessed the expression level of individual mRNAs by counting the number of reads that aligned uniquely to each gene, and then normalizing this number to allow comparisons across all samples.

To determine the amount of sequencing reads that came from each virus, we aligned the reads to the Lassa virus and Marburg virus genomes. In the Lassa infection group, only the samples taken 10 days post-infection (dpi) contained viral reads (less than 20 reads) (Figure 1B). In the Marburg infection group, around 2000 viral reads were seen 5 dpi, and around 80,000 reads 9 dpi (Figure 1C). These numbers suggest that peripheral blood mononuclear cells are early targets for Marburg virus but not for Lassa virus. The number of viral reads found in PBMCs correlates well with the level of viremia, as previously reported for Lassa virus [18] and Marburg virus [Lee et al., manuscript in preparation].

### The common transcriptional response of PBMCs during Lassa and Marburg infection is dominated by type I interferon-stimulated genes

**Figure 2:**
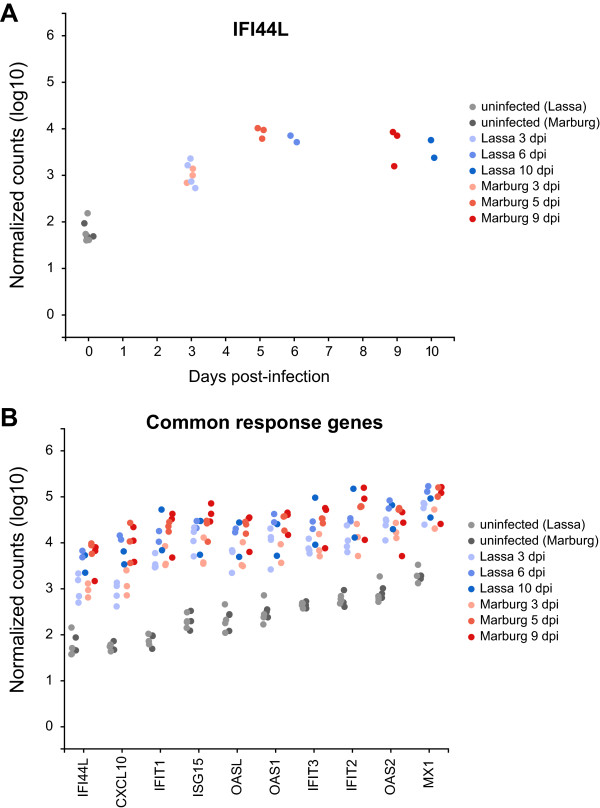
**Genes showing similar patterns of expression during Lassa virus and Marburg virus infection.** Each point represents the level of expression (measured in log_10_ normalized read counts) of a gene that behaves similarly in both types of infection (increasingly darker shades of blue for Lassa, and red for Marburg). Panel **(A)** represents the expression of gene IFI44L showing time in the x-axis. Panel **(B)** condenses the information in **(A)** and shows additional genes along the x-axis.

To identify what transcriptional changes are shared by different hemorrhagic fever virus infections, we identified genes whose levels of expression 1) closely matched in both types of viral challenge, 2) strongly increased or decreased 3 days post-infection (dpi), and 3) increased or sustained this level of expression throughout the remaining time points (5–10 dpi). We labeled this group of genes the *common response* (Figure 2B). Some of its representative members include transcription factors IRF7 and STAT1, which serve as master regulators of host immunity; pattern recognition receptors DDX58 (RIG-I), IFIH1 (MDA-5) and DHX58 (LGP2), which activate different signaling cascades in the innate immune system; type I interferon-stimulated genes ISG15, ISG20, OAS1, OAS2, OASL, MX1, IFIT1, IFIT2, IFIT3, HERC5, HERC6, IFI6, IFI35, IFI44 and IFI44L, which play a variety of antiviral roles [22]; and the cytokine CXCL10, which attracts activated T cells [23] (Additional file 1).

### A subset of genes shows unique patterns of expression in each type of infection

After identifying the subset of genes that made up the common transcriptional response, we looked for genes that showed unique transcriptional patterns. To do this, we considered genes that 1) showed a statistically significant increase or decrease in their 3 dpi levels of expression for one type of viral infection but not the other, and 2) increased or sustained this difference in expression throughout the later stages of the infection.

**Figure 3:**
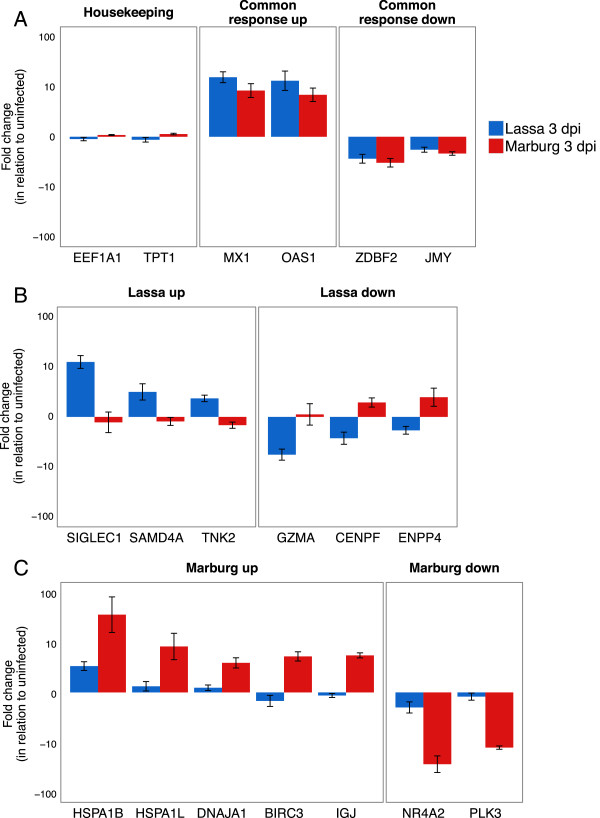
**Gene expression patterns during Lassa or Marburg virus infection.** A few representative genes are shown in each panel representing each category: **A)** housekeeping genes, genes that uniformly increase or decrease their expression 3 days after both infections, **B)** genes that show unique patterns of expression during Lassa infection, and **C)** during Marburg infection. The y-axis represents the average fold change in expression when comparing infected and uninfected samples.

In the Lassa infection group, we detected several genes with distinctly upregulated expression (Figure 3 and Additional file 1), including SIGLEC1, a cell adhesion molecule expressed by dendritic cells and macrophages [24, 25]; SAMD4, a translational repressor [26]; and TNK2, a tyrosine kinase implicated in cell spreading and migration [27]. Other genes, like CENPF, ENPP4, and GZMA, a protease responsible for the release of cytotoxic T-cell granules [28], were significantly downregulated during Lassa infection in comparison to Marburg infection.

In the Marburg infection group, we identified a distinct upregulation in the expression of genes encoding heat shock proteins (HSPA1B, HSPA1L and DNAJA1), the antiapoptotic gene BIRC3, and the immunoglobulin genes IGJ, IGLV10-54, among others. We also found genes that showed lower levels of expression during Marburg infection when compared to Lassa infection. Examples of these include PLK3, a kinase associated with stress response and apoptosis [29, 30]; and NR4A2, a nuclear receptor which has been shown to induce apoptosis when downregulated [31]. The patterns of expression for these and other genes are shown in Additional files 2 and 3.

### Validation of infection-specific patterns of expression using microarrays and RT-PCR

We wanted to know if the genes that showed significant differences in each type of infection could be used to classify unknown samples. Although we were not able to obtain an independent test dataset, we used additional samples from two previous studies to characterize the host transcriptional response to Lassa [18] and Marburg infection [Lee et al., manuscript in preparation] (Additional files 4 and 5). This dataset included the 24 samples that we measured using RNA sequencing, as well as 26 additional uninfected samples, 12 Lassa-infected samples and 3 Marburg-infected samples (Figure 1A), for a total of 65 samples, all of which were quantified using Agilent two-color human microarrays. We looked at the genes that we had previously identified (using RNA sequencing) as being differentially expressed during one of the two infections, and found that the measurements from both platforms were highly correlated (0.81 Pearson’s correlation).

**Figure 4:**
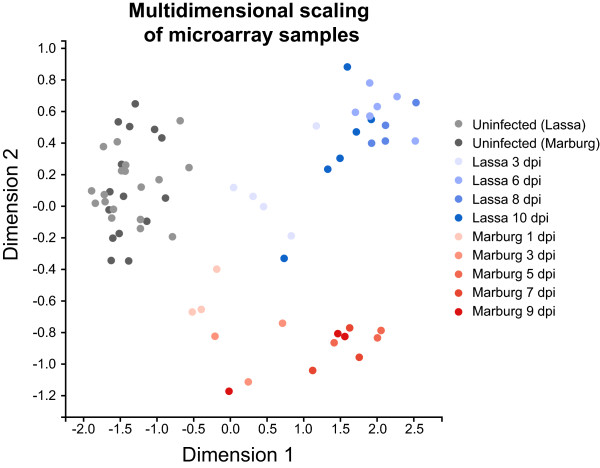
**Clustering of microarray samples.** Each point corresponds to an RNA sample, and their distances represent how similar they are based on applying Multidimensional Scaling to the microarray expression values of 16 genes: 4 common response genes (MX1, OAS1, IFI44L and CXCL10), 4 Marburg-specific response genes (HSPA1B, IGJ, HSPA1L and BIRC3) and 4 Lassa-specific response genes (SIGLEC1, TNK2, TNFSF10 and NR4A2). Lassa- and Marburg-infected samples are shown in increasingly darker shades of blue and red, respectively.

**Figure 5:**
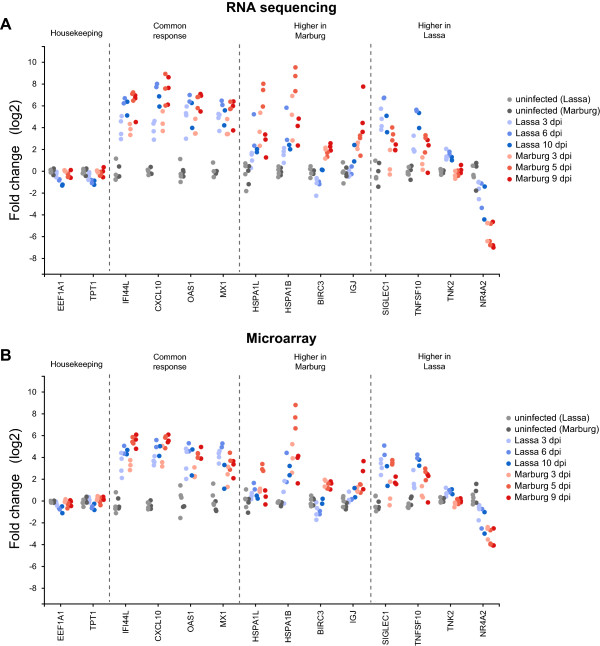
**Biomarker genes quantified using RNA sequencing and microarrays.** For each gene, the y-axis represents its amount of expression in each RNA sample, and the x-axis represents these samples ordered by time of infection, colored in increasingly darker shades of blue for Lassa, and red for Marburg. The samples in **(A)** were quantified using RNA sequencing and the fold change represents the log_2_ difference between the average infected and uninfected normalized read counts. The samples in **(B)** were measured using two-color microarrays and the fold change represents the log_2_ ratio between the intensity of the red channel and the green channel (see Methods).

**Figure 6:**
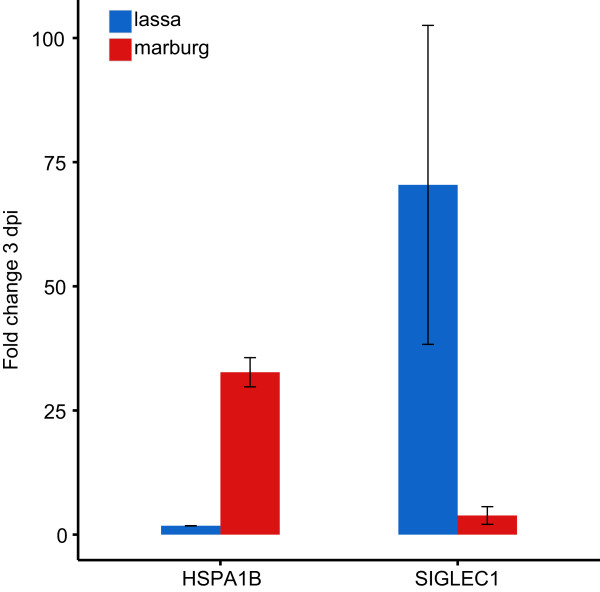
**3 dpi RT-PCR validation.** SIGLEC1 and HSPA1B are the genes with the highest fold change differences between each virus group 3 days post-infection. The y-axis represents the difference in expression between each gene and the constitutively expressed gene BIN2. The RT-PCR reactions for each gene were performed in duplicate (n = 2).

To understand in more detail the differences in expression reported by RNA sequencing and microarrays, we classified a subset of genes into four categories: 1) **Housekeeping genes**, those that showed high levels of expression across all samples. 2) **Common response genes**, those where infected samples show higher levels of expression than uninfected samples, and where these levels of expression were similar in both types of infection. 3) **Marburg-specific response genes**, those where Marburg-infected samples showed significantly higher (or lower) patterns of expression when compared to uninfected or Lassa-infected samples. 4) **Lassa-specific response genes**, those where Lassa-infected samples showed significantly higher patterns of expression when compared to uninfected or Marburg-infected samples.To determine if we could use the genes showing unique regulation at early times of infection to distinguish between different types of infected samples, we applied Multidimensional Scaling (MDS)—a dimensionality reduction technique similar to Primary Component Analysis—to the microarray samples. Instead of applying MDS on the expression of every gene, we chose a subset of common response genes, Marburg-specific response genes and Lassa-specific response genes (see Figure 4). Reducing the dimensionality of all samples using these genes resulted in three clear clusters: uninfected samples, infected with Lassa virus, and infected with Marburg virus. Each of the infected clusters contained not only the early-infected samples, but also those samples taken at later stages of infection. This indicates that the expression patterns of these genes are useful indicators throughout both early and late stages of infection.We then looked at the reported expression levels for these genes in both platforms (Figure 5). We only used samples that were quantified in both sequencing and arrays to ensure that differences in variability would only be related to the platform, not to the number of samples. The majority of genes showed similar levels of expression in both platforms. The biggest differences came from genes like HSPA1L and IGJ, which showed much smaller fold changes in arrays than in sequencing. On average, both highly upregulated and downregulated genes showed fold changes with a 1.5-2 times smaller magnitude in arrays when compared to sequencing.This comparison confirmed that both RNA sequencing and microarrays show unique changes in mRNA levels early after infection. This led us to test the hypothesis that a more direct RT-PCR analysis of a small number of these sentinel genes would show similar patterns of unique regulation, with the advantage that the format of this assay could be applied more quickly and cheaply in the field. We chose to analyze the expression of two genes, SIGLEC1 and HSPA1B, which showed unique upregulation at early stages of Lassa or Marburg virus infection, respectively. RT-PCR assays carried out on uninfected samples, and on samples collected 3 days post-infection, showed that there was no statistically significant change in SIGLEC1 expression between these two time points in Marburg-infected animals, but that there was a 70-fold change in expression in Lassa-infected animals. Similarly, HSPA1B showed a minor change in Lassa-infected animals, but a 36-fold change in Marburg-infected animals. This confirmed that SIGLEC1 becomes upregulated during the early stages of Lassa infection but not Marburg infection, while HSPA1B is only upregulated during the early stages of Marburg infection (Figure 6).

## Discussion

In the work described in this manuscript, we investigated the hypothesis that the circulating immune response can be used to aid early diagnosis of viral infections that are associated with high levels of mortality. When we analyzed the transcriptional patterns of the circulating immune system in response to two different hemorrhagic fever viruses, we identified a subset of genes that uniquely increased their rate of transcription 3 days after infection and that sustained these differences throughout infection (Figure 3).

An important aspect of this study was that the uniquely regulated genes were not the only genes that changed their patterns of expression during the early times of infection (before the detection of clinical symptoms), but that many other genes—pattern recognition receptors, interferon-stimulated genes, cytokines, and immune and antiviral transcriptional regulators—also became rapidly upregulated. This suggests that early after infection, there is a strong activation of the type I interferon innate immune response. This early innate response has also been reported for infections with other viruses, such as Ebola [32], influenza [33] and respiratory syncytial virus (RSV) [34], and suggests that the activation of the innate immune system is being driven by a different mechanism. Surprisingly, we did not detect a significant upregulation of type I interferon genes, although we did see it for their downstream effectors. Protein studies indicate that type I interferon is present during Marburg virus infection, suggesting that interferon transcription might be localized to plasmacytoid dendritic cells, which are not strongly represented in the PBMC population [35].

In addition to the common transcriptional changes that we observed at early times of infection, we also identified a number of genes with unique patterns of expression in each type of infection. The unique markers of Marburg infection were generally classifiable into functional categories, including heat-shock proteins (HSPA1B, HSPA1L, DNAJA1) and immunoglobulin-associated genes (IGJ, IGLV10-54). These results suggest that the response to Marburg infection leads to the expansion of both stress adaptive and immunological maturation pathways at very early times of infection.

Some of the most uniquely expressed genes during Lassa infection are SIGLEC1 (also known as CD169 or sialoadhesin) and SAMD4A. The upregulation of these genes suggests an increase in SIGLEC1+ SAMD4A + macrophages early in Lassa infection [25, 36]. A more detailed understanding of the mechanisms leading to the unique signatures identified will require further investigation. While both Lassa virus and Marburg virus are known to evade antiviral responses by inhibiting the expression of specific host genes [37, 38], it is unlikely that these mechanisms are directly responsible for the expression differences that we found in PBMCs, since they take place during the pre-viremic stages of infection. The specific differences observed take place even in the absence of any viral genetic material in the PBMCs, which suggests that these circulating cells are responding to an external signal. An alternative explanation for the existence of unique signatures might be related to each pathogen’s cell tropism: Marburg virus preferentially targets monocytes, macrophages, dendritic cells and Kupffer cells [39], and leads to significant lymphocyte apoptosis [40]. Lassa virus targets dendritic cells, Kupffer cells, hepatocytes, adrenal cortical cells and endothelial cells [41], and is not known to induce lymphocyte decreases. It is possible that a combination of which cells become initially infected and the magnitude of the early response combine to ultimately produce the infection-specific signatures that we observe.

We have shown that microarrays can be used to validate the changes in expression that take place in Lassa- and Marburg-infected PBMCs in addition to RNA sequencing. Although the majority of genes that undergo significant early changes in expression were highly correlated in both platforms, microarrays showed a smaller range of gene expression differences between both types of infected samples and greater variability. Three previous studies that analyzed the immune response to Lassa virus infection [18, 42] and LCMV virus infection [43] were in general agreement with the major trends that we identified using RNA sequencing: mainly the upregulation of interferon-stimulated genes, pattern recognition receptors and pro-inflammatory cytokines.

The infection-specific transcriptional changes that we have found suggest a method for discriminating between diseases with similar early clinical symptoms, as is the case for Lassa and Marburg infection. In infected animals, these transcriptional changes take place prior to the appearance of viremia and other overt symptoms [18], and they remain sustained throughout the late stages of infection, thereby providing a way to diagnose which virus is most likely causing the infection. This diagnosis can be carried out during the early time window when treatment is most effective, and before the pathogen starts replicating in the bloodstream on a massive scale. To ensure that an RT-PCR-based diagnostic would be able to distinguish between diseases other than Lassa and Marburg disease, it would be important to determine the host transcriptional response to additional pathogens like influenza virus, dengue virus or the malaria parasite, which cause similar initial symptoms to those of viral hemorrhagic fevers.

## Conclusions

The expression of a unique set of host genes increases or decreases preferentially in Lassa- or Marburg-infected immune cells. During Marburg virus infection, heat-shock protein and immunoglobulin genes show significantly greater levels of expression than during Lassa virus infection, which is characterized by the upregulation of the cell adhesion molecule SIGLEC1, the translational repressor SAMD4A and the tyrosine kinase TNK2. These changes in expression appear early after infection and remain sustained throughout the entire course of disease, which provides a way to diagnose which virus is most likely to be causing the infection without having to wait until the appearance of viremia or other overt symptoms. The fact that these changes were identified using multiple platforms (RNA sequencing, microarrays and RT-PCR) suggests a path for translating these gene expression profiles into a diagnostic test with direct clinical applications.

## Methods

### Animals

The samples used in this study came from a larger sequential sampling study designed to characterize the disease progression of viral hemorrhagic fevers after aerosol exposure [Honko et al*.*, manuscript in preparation].

#### Ethics statement

Animal studies were carried out in accordance with standards of IACUC approved protocol in compliance with the regulations outlined in the USDA Animal Welfare Act (PHS Policy), and other Federal statutes and regulations relating to experiments involving animals. The facility where this research was conducted is accredited by the Association for Assessment and Accreditation of Laboratory Animal Care International and all animal work adheres to the conditions specified in the *Guide for the Care and Use of Laboratory Animals* (National Research Council, [44]). These experiments and procedures were approved by the USAMRIID Animal Care and Use Committee (IACUC). Animals were given enrichment regularly as recommended by the *Guide for the Care and Use of Laboratory Animals*. The animals were fed and checked at least daily according to the protocol. All efforts were made to minimize painful procedures; the attending veterinarian was consulted regarding painful procedures, and animals were anesthetized prior to phlebotomy. Following the development of clinical signs, animals were checked multiple times daily. When clinical observations and scores reached defined levels based on the approved IACUC Animal Care and Use Committee protocol, the animals were euthanized under anesthesia to minimize pain and distress.

#### LASV sequential sampling study

For this study, seven adult male and female cynomolgus macaques (*Macaca fascicularis*), ranging in weight from 5.5 to 8.7 kg were obtained from licensed and approved vendors. During the acclimation to the biosafety level 4 (BSL4) containment suite, a pre-challenge blood sample (8 days before exposure) was collected to provide a baseline for further analyses. (These samples are referred to in this manuscript as pre-infection or 0 days post-infection samples.) On day 0, NHPs were anesthetized and minute volumes calculated by performing whole-body plethysmography (Buxco Research Systems, Wilmington, NC) just prior to the exposure. NHPs were exposed individually at a target dose of 1000 Plaque Forming Unit (PFU) of LASV Josiah in a head-only chamber in a class III biological safety cabinet maintained under negative pressure within a BSL-4 laboratory. Aerosols were created by a 3-jet Collison nebulizer (BGI Inc., Waltham, MA) and controlled by the USAMRIID Automated Bioaerosol Exposure System (ABES-II). The virus preparation used to infect NHPs was free of contamination of endotoxin and mycoplasma. After challenge, blood samples were collected at various days post-infection (dpi), based on approved collection allowances, as well as at euthanasia. Groups of two to three NHP were euthanized at 3, 6, 8 and 10 dpi, and peripheral blood mononuclear cells (PBMCs) were prepared from collected blood.

#### MARV sequential sampling study

The Marburg study was performed under the same conditions as the Lassa study, with three differences:

1) The virus strain used was MARV Angola, 2) the number of adult male and female macaques was nine, and 3) macaques were euthanized and PBMCs collected from blood at 1, 3, 5, 7 and 9 dpi.

#### RNA processing

PBMCs were isolated from blood pre-diluted with saline using ACCUSPIN System-Histopaque-1077 tubes as per manufacturer’s recommendations, and subsequently lysed in TRI Reagent LS (Sigma-Aldrich, St. Louis, MO) at USAMRIID.

### Next-generation sequencing

#### Library preparation

A subset of the total RNA samples from both datasets was processed for RNA sequencing (Figure 1A). Sequencing libraries were prepared for mRNA sequencing using TruSeq v2 RNA Sample Preparation kits (Illumina, San Diego, CA). Briefly, per the manufacturer protocol, mRNA was purified from approximately 500 ng to 1 μg of total RNA per sample using poly-T oligo-attached magnetic beads, then fragmented and primed with random hexamers for cDNA synthesis. Multiple indexing adapters were ligated to the ends of the double-stranded cDNA, and the DNA fragments with adapter molecules on both ends were then selectively PCR enriched to create the sequencing libraries. Libraries were validated for size and purity on an Agilent 2100 Bioanalyzer using the DNA-1000 chip (Agilent Technologies, Santa Clara, CA) before they were normalized and pooled for multiplex sequencing. Cluster generation and sequencing were performed by the Analytical Core Facility at Tufts University (Department of Physiology).

#### Fold-change analysis

After sequencing the *Macaca fascicularis* PBMC RNA samples from both types of infection (Lassa and Marburg), the generated reads were trimmed and the adapters were removed using Trimmomatic [45]. The processed reads were aligned to the *Macaca mulatta* genome (Ensembl release 75) using TopHat 2.0.6 [46] with default parameters (segment length of 25, allowing up to 2 segment mismatches) and using the RhesusBase2 [47] annotated *M. mulatta* transcriptome as reference. Gene counts were obtained using HTSeq [48] to count reads that aligned uniquely to each gene. Counts were normalized to compensate for differences in library size using the trimmed mean of M-values normalization method [49] included in the edgeR BioConductor package [50]. The normalized sequenced reads were also aligned to the Lassa virus (NCBI BioProject accession PRJNA14864) and Marburg virus (NCBI BioProject accession PRJNA15199) genomes using BWA [51] to quantify the number of viral reads in each sample.

A gene was deemed to show statistically significant changes in expression at a specific time after infection if the moderated t-test [50] between the infected and pre-infection samples resulted in a multiple testing-corrected p-value lower than 0.05, with an absolute fold change in expression greater or equal to 3, and an average number of reads across all samples greater than 4 counts per million (CPM). When calculating relative changes to the pre-infection samples, infected samples were not subtracted from their individual uninfected controls (since not every infected sample had a pre-infected control), but from an average of all the pre-infection samples. Statistical significance, however, was calculated using the individual pre-infection samples, not their average.

### DNA microarrays

#### Sample preparation

PBMCs were processed for microarray analysis as described previously [52]. Briefly, total RNA was extracted from the TRI Reagent LS samples, then amplified using the Low-Input Quick Amp Labeling kit (Agilent Technologies, Santa Clara, CA) and hybridized to Whole Human Genome Oligo Microarrays (Agilent Technologies, Santa Clara, CA) in a 2-color comparative format along with a reference pool of messenger RNA. Images were scanned using the Agilent High-Resolution Microarray Scanner and raw microarray images were processed using Agilent’s Feature Extraction software.

#### Fold-change analysis

Agilent two-color human gene expression microarrays were processed using limma [53]. Fold changes were obtained by calculating the log-ratio between the intensities of the red channel (which corresponds to experimental samples) and the green channel (which corresponds to Human Universal Reference RNA [54]). These were later background-corrected and normalized using LOESS [55].

### Multidimensional scaling

The limma [53] implementation of Multidimensional Scaling was used to reduce the dimensionality of a matrix composed of the expression values of the following 16 genes across the 66 RNA samples: MX1, OAS1, IFI44L, CXCL10, HSPA1B, IGJ, HSPA1L, BIRC3, SIGLEC1, TNK2, TNFSF10, NR4A2.

### Reverse transcriptase polymerase chain reaction

RT-PCR reactions were carried out in 50 μL reactions using Power SYBR® Green RNA-to-CT™ 1-Step Kit from Life Technologies: 25 μL SYBR® Green RT-PCR Mix, 2 μL of each primer at 100 μM, and .4 μL RT Enzyme Mix. Thermal cycling was conducted as follows: 30 minutes at 48°C, 10 minutes at 95°C, 15 seconds at 95°C (40 cycles), and 1 minute at 60°C (40 cycles). The primer sequences used were: SIGLEC1 (FWD) 5′-TCCTGAGTGTACTCTATCCC-3′ (REV) 5′-TCGACAGTGCAGACAAAC-3′; BIRC3 (FWD) 5′-ACTTGAACAGCTGCTATCT-3′ (REV) 5′-TCTTCTGAATGGTCTTCTCC-3′; BIN2 (FWD) 5′-CTCCTGAGGCCAAAGAAA-3′ (REV) 5′-AGGCTCTGAAGCAATCTG-3′. BIN2 was chosen as the control gene because sequencing analysis showed it was highly expressed across all samples and had a small relative standard deviation.

### Availability of supporting data

The sequencing data sets supporting the results of this article are available in the Sequence Read Archive (SRA) under accession numbers PRJNA222891 (Lassa) and PRJNA222892 (Marburg). The microarray datasets are available in the Gene Expression Omnibus (GEO) database, under series record GSE41752 (LASV) [18] and GSE58287 (MARV) [Lee et al., manuscript in preparation].

## Electronic supplementary material

Additional file 1: **List of genes categorized as common response, more highly expressed in Marburg, or more highly expressed in Lassa.** Columns include the Ensembl gene ids, gene names, and the corresponding category. (CSV 3 KB)

Additional file 2: **Visual representation of genes categorized as more highly expressed in Lassa.** The x-axis represents time and the y-axis represents normalized read counts. (PDF 15 KB)

Additional file 3: **Visual representation of genes categorized as more highly expressed in Marburg.** The x-axis represents time and the y-axis represents normalized read counts. (PDF 11 KB)

Additional file 4: **Table of gene normalized read counts during Lassa infection.** Columns include the Ensembl gene ids, gene names, and the mean normalized read counts and standard deviations for each timepoint. (CSV 2 MB)

Additional file 5: **Table of gene normalized read counts during Marburg infection.** Columns include the Ensembl gene ids, gene names, and the mean normalized read counts and standard deviations for each timepoint. (CSV 2 MB)

## References

[1] McCormick JB, King IJ, Webb P a, Johnson KM, O’Sullivan R, Smith ES, Trippel S, Tong TC (1987). A case–control study of the clinical diagnosis and course of Lassa fever. J Infect Dis.

[2] Macher A, Wolfe M (2006). Historical Lassa fever reports and 30-year clinical update. Emerg Infect Dis.

[3] Buckley SM, Casals J (1970). Lassa fever, a new virus disease of man from West Africa. 3. Isolation and characterization of the virus. Am J Trop Med Hyg.

[4] Shaffer JG, Grant DS, Schieffelin JS, Boisen ML, Goba A, Hartnett JN, Levy DC, Yenni RE, Moses LM, Fullah M, Momoh M, Fonnie M, Fonnie R, Kanneh L, Koroma VJ, Kargbo K, Ottomassathien D, Muncy IJ, Jones AB, Illick MM, Kulakosky PC, Haislip AM, Bishop CM, Elliot DH, Brown BL, Zhu H, Hastie KM, Andersen KG, Gire SK, Tabrizi S (2014). Lassa fever in post-conflict sierra leone. PLoS Negl Trop Dis.

[5] Bausch DG, Nichol ST, Muyembe-Tamfum JJ, Borchert M, Rollin PE, Sleurs H, Campbell P, Tshioko FK, Roth C, Colebunders R, Pirard P, Mardel S, Olinda LA, Zeller H, Tshomba A, Kulidri A, Libande ML, Mulangu S, Formenty P, Grein T, Leirs H, Braack L, Ksiazek T, Zaki S, Bowen MD, Smit SB, Leman PA, Burt FJ, Kemp A, Swanepoel R (2006). Marburg hemorrhagic fever associated with multiple genetic lineages of virus. N Engl J Med.

[6] Towner JS, Khristova ML, Sealy TK, Vincent MJ, Erickson BR, Bawiec DA, Hartman AL, Comer JA, Zaki SR, Ströher U, Gomes da Silva F, del Castillo F, Rollin PE, Ksiazek TG, Nichol ST (2006). Marburgvirus genomics and association with a large hemorrhagic fever outbreak in Angola. J Virol.

[7] Timen A (2009). Response to imported case of marburg hemorrhagic fever, the Netherlands. Emerg Infect Dis.

[8] Fujita N, Miller A, Miller G, Gershman K, Gallagher N, Marano N, Hale C, Jentes E (2009). Imported case of Marburg hemorrhagic fever - Colorado, 2008. MMWR Morb Mortal Wkly Rep.

[9] Drosten C, Kümmerer B, Schmitz H, Günther S (2003). Molecular diagnostics of viral hemorrhagic fevers. Antiviral Res.

[10] Green A (2014). West Africa struggles to contain Ebola outbreak. Lancet.

[11] Baize S, Pannetier D, Oestereich L, Rieger T, Koivogui L, Magassouba N, Soropogui B, Sow MS, Keïta S, De Clerck H, Tiffany A, Dominguez G, Loua M, Traoré A, Kolié M, Malano ER, Heleze E, Bocquin A, Mély S, Raoul H, Caro V, Cadar D, Gabriel M, Pahlmann M, Tappe D, Schmidt-Chanasit J, Impouma B, Diallo AK, Formenty P, Van Herp M (2014). Emergence of Zaire Ebola virus disease in Guinea - preliminary report. N Engl J Med.

[12] Panning M, Emmerich P, Olschläger S, Bojenko S, Koivogui L, Marx A, Lugala PC, Günther S, Bausch DG, Drosten C (2010). Laboratory diagnosis of Lassa fever, liberia. Emerg Infect Dis.

[13] Bausch DG, Rollin PE, Demby AH, Coulibaly M, Kanu J, Conteh AS, Wagoner KD, McMullan LK, Bowen MD, Peters CJ, Ksiazek TG (2000). Diagnosis and clinical virology of Lassa fever as evaluated by enzyme-linked immunosorbent assay, indirect fluorescent-antibody test, and virus isolation. J Clin Microbiol.

[14] Grolla A, Lucht A, Dick D, Strong JE, Feldmann H (2005). Laboratory diagnosis of Ebola and Marburg hemorrhagic fever. Bull Soc Pathol Exot.

[15] Jahrling PB, Peters CJ, Stephen EL (1984). Enhanced treatment of Lassa fever by immune plasma combined with ribavirin in cynomolgus monkeys. J Infect Dis.

[16] Geisbert TW, Feldmann H (2011). Recombinant vesicular stomatitis virus-based vaccines against Ebola and Marburg virus infections. J Infect Dis.

[17] Ursic-Bedoya R, Mire CE, Robbins M, Geisbert JB, Judge A, Maclachlan I, Geisbert TW (2014). Protection against lethal Marburg virus infection mediated by lipid encapsulated small interfering RNA. J Infect Dis.

[18] Malhotra S, Yen JY, Honko AN, Garamszegi S, Caballero IS, Johnson JC, Mucker EM, Trefry JC, Hensley LE, Connor JH (2013). Transcriptional profiling of the circulating immune response to Lassa virus in an aerosol model of exposure. PLoS Negl Trop Dis.

[19] Baas T, Baskin CR, Diamond DL, García-Sastre A, Bielefeldt-Ohmann H, Tumpey TM, Thomas MJ, Carter VS, Teal TH, Van Hoeven N, Proll S, Jacobs JM, Caldwell ZR, Gritsenko MA, Hukkanen RR, Camp DG, Smith RD, Katze MG (2006). Integrated molecular signature of disease: analysis of influenza virus-infected macaques through functional genomics and proteomics. J Virol.

[20] Zaas AK, Burke T, Chen M, McClain M, Nicholson B, Veldman T, Tsalik EL, Fowler V, Rivers EP, Otero R, Kingsmore SF, Voora D, Lucas J, Hero AO, Carin L, Woods CW, Ginsburg GS (2013). A host-based RT-PCR gene expression signature to identify acute respiratory viral infection. Sci Transl Med.

[21] Hu X, Yu J, Crosby SD, Storch G a (2013). Gene expression profiles in febrile children with defined viral and bacterial infection. Proc Natl Acad Sci U S A.

[22] Schoggins JW, Wilson SJ, Panis M, Murphy MY, Jones CT, Bieniasz P, Rice CM (2011). A diverse range of gene products are effectors of the type I interferon antiviral response. Nature.

[23] Dufour JH, Dziejman M, Liu MT, Leung JH, Lane TE, Luster AD (2002). IFN-gamma-inducible protein 10 (IP-10; CXCL10)-deficient mice reveal a role for IP-10 in effector T cell generation and trafficking. J Immunol.

[24] Izquierdo-Useros N, Lorizate M, Puertas MC, Rodriguez-Plata MT, Zangger N, Erikson E, Pino M, Erkizia I, Glass B, Clotet B, Keppler OT, Telenti A, Kräusslich H-G, Martinez-Picado J (2012). Siglec-1 is a novel dendritic cell receptor that mediates HIV-1 trans-infection through recognition of viral membrane gangliosides. PLoS Biol.

[25] Crocker PR, Paulson JC, Varki A (2007). Siglecs and their roles in the immune system. Nat Rev Immunol.

[26] Baez MV, Boccaccio GL (2005). Mammalian Smaug is a translational repressor that forms cytoplasmic foci similar to stress granules. J Biol Chem.

[27] Howlin J, Rosenkvist J, Andersson T (2008). TNK2 preserves epidermal growth factor receptor expression on the cell surface and enhances migration and invasion of human breast cancer cells. Breast Cancer Res.

[28] Hink-Schauer C, Estébanez-Perpiñá E, Kurschus FC, Bode W, Jenne DE (2003). Crystal structure of the apoptosis-inducing human granzyme A dimer. Nat Struct Biol.

[29] Bahassi EM, Conn CW, Myer DL, Hennigan RF, McGowan CH, Sanchez Y, Stambrook PJ (2002). Mammalian Polo-like kinase 3 (Plk3) is a multifunctional protein involved in stress response pathways. Oncogene.

[30] Xie S, Wu H, Wang Q, Cogswell JP, Husain I, Conn C, Stambrook P, Jhanwar-Uniyal M, Dai W (2001). Plk3 functionally links DNA damage to cell cycle arrest and apoptosis at least in part via the p53 pathway. J Biol Chem.

[31] Ke N, Claassen G, Yu D-H, Albers A, Fan W, Tan P, Grifman M, Hu X, Defife K, Nguy V, Meyhack B, Brachat A, Wong-Staal F, Li Q-X (2004). Nuclear hormone receptor NR4A2 is involved in cell transformation and apoptosis. Cancer Res.

[32] Gupta M, Mahanty S, Ahmed R, Rollin PE (2001). Monocyte-derived human macrophages and peripheral blood mononuclear cells infected with ebola virus secrete MIP-1alpha and TNF-alpha and inhibit poly-IC-induced IFN-alpha in vitro. Virology.

[33] Huang Y, Zaas AK, Rao A, Dobigeon N, Woolf PJ, Veldman T, Øien NC, McClain MT, Varkey JB, Nicholson B, Carin L, Kingsmore S, Woods CW, Ginsburg GS, Hero AO (2011). Temporal dynamics of host molecular responses differentiate symptomatic and asymptomatic influenza a infection. PLoS Genet.

[34] Chen M, Carlson D, Zaas A, Woods CW, Ginsburg GS, Hero A, Lucas J, Carin L (2011). Detection of viruses via statistical gene expression analysis. IEEE Trans Biomed Eng.

[35] Fritz E a, Geisbert JB, Geisbert TW, Hensley LE, Reed DS (2008). Cellular immune response to Marburg virus infection in cynomolgus macaques. Viral Immunol.

[36] Van der Kuyl AC, van den Burg R, Zorgdrager F, Groot F, Berkhout B, Cornelissen M (2007). Sialoadhesin (CD169) expression in CD14+ cells is upregulated early after HIV-1 infection and increases during disease progression. PLoS ONE.

[37] Hayes M, Salvato M (2012). Arenavirus evasion of host anti-viral responses. Viruses.

[38] Basler CF, Amarasinghe GK (2009). Evasion of interferon responses by Ebola and Marburg viruses. J Interferon Cytokine Res.

[39] Hensley LE, Alves DA, Geisbert JB, Fritz EA, Reed C, Larsen T, Geisbert TW (2011). Pathogenesis of Marburg hemorrhagic fever in cynomolgus macaques. J Infect Dis.

[40] Geisbert TW, Hensley LE, Gibb TR, Steele KE, Jaax NK, Jahrling PB (2000). Apoptosis induced in vitro and in vivo during infection by Ebola and Marburg viruses. Lab Invest.

[41] Hensley LE, Smith MA, Geisbert JB, Fritz EA, Daddario-DiCaprio KM, Larsen T, Geisbert TW (2011). Pathogenesis of Lassa fever in cynomolgus macaques. Virol J.

[42] Zapata JC, Carrion R, Patterson JL, Crasta O, Zhang Y, Mani S, Jett M, Poonia B, Djavani M, White DM, Lukashevich IS, Salvato MS (2013). Transcriptome analysis of human peripheral blood mononuclear cells exposed to Lassa virus and to the attenuated Mopeia/Lassa reassortant 29 (ML29), a vaccine candidate. PLoS Negl Trop Dis.

[43] Djavani MM, Crasta OR, Zapata JC, Fei Z, Folkerts O, Sobral B, Swindells M, Bryant J, Davis H, Pauza CD, Lukashevich IS, Hammamieh R, Jett M, Salvato MS (2007). Early blood profiles of virus infection in a monkey model for Lassa fever. J Virol.

[44] National Research Council of the National Academies (2011). Guide for the care and use of laboratory animals.

[45] Bolger AM, Lohse M, Usadel B (2014). Trimmomatic: a flexible trimmer for Illumina sequence data. Bioinformatics.

[46] Trapnell C, Pachter L, Salzberg SL (2009). TopHat: discovering splice junctions with RNA-Seq. Bioinformatics.

[47] Zhang S-J, Liu C-J, Shi M, Kong L, Chen J-Y, Zhou W-Z, Zhu X, Yu P, Wang J, Yang X, Hou N, Ye Z, Zhang R, Xiao R, Zhang X, Li C-Y (2013). RhesusBase: a knowledgebase for the monkey research community. Nucleic Acids Res.

[48] Anders S (2010). HTSeq: Analysing high-throughput sequencing data with Python.

[49] Robinson MD, Oshlack A (2010). A scaling normalization method for differential expression analysis of RNA-seq data. Genome Biol.

[50] Robinson MD, McCarthy DJ, Smyth GK (2010). edgeR: a Bioconductor package for differential expression analysis of digital gene expression data. Bioinformatics.

[51] Li H, Durbin R (2009). Fast and accurate short read alignment with Burrows-Wheeler transform. Bioinformatics.

[52] Yen JY, Garamszegi S, Geisbert JB, Rubins KH, Geisbert TW, Honko A, Xia Y, Connor JH, Hensley LE (2011). Therapeutics of Ebola hemorrhagic fever: whole-genome transcriptional analysis of successful disease mitigation. J Infect Dis.

[53] Smyth GK (2004). Linear models and empirical bayes methods for assessing differential expression in microarray experiments. Stat Appl Genet Mol Biol.

[54] Novoradovskaya N, Whitfield ML, Basehore LS, Novoradovsky A, Pesich R, Usary J, Karaca M, Wong WK, Aprelikova O, Fero M, Perou CM, Botstein D, Braman J (2004). Universal Reference RNA as a standard for microarray experiments. BMC Genomics.

[55] Smyth GK, Speed T (2003). Normalization of cDNA microarray data. Methods.

