# Supplementary figures and images for: Lassa and Marburg viruses elicit distinct host transcriptional responses early after infection
Source: BMC Genomics. 2014 Nov 6;15(1):960. doi: 10.1186/1471-2164-15-960 (PMC4232721; doi:10.1186/1471-2164-15-960)

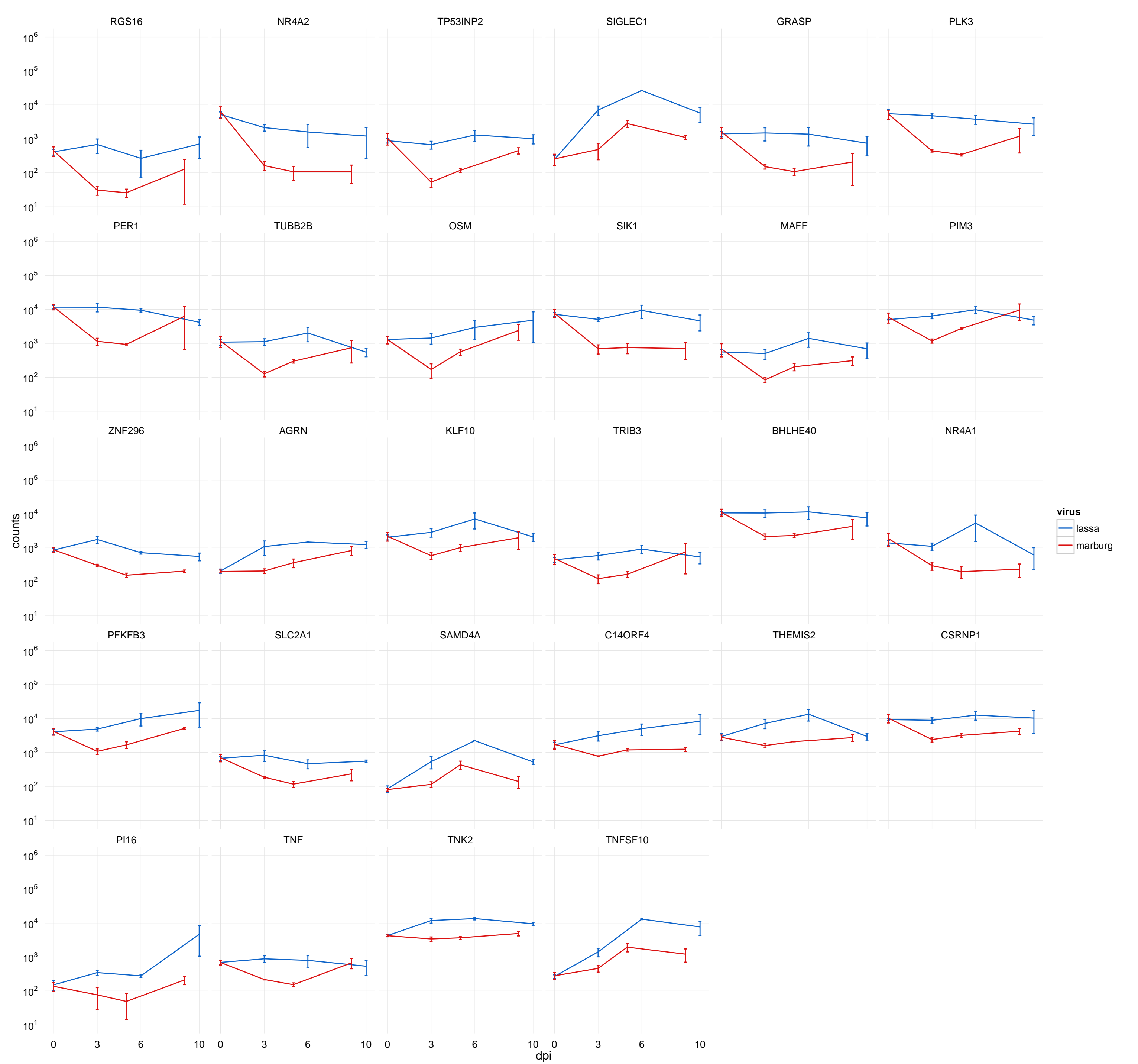

Supplement: Supplementary file 2 — Additional file 2: Visual representation of genes categorized as more highly expressed in Lassa. The x-axis represents time and the y-axis represents normalized read counts. (PDF 15 KB) [file 12864_2014_6648_MOESM2_ESM.pdf]

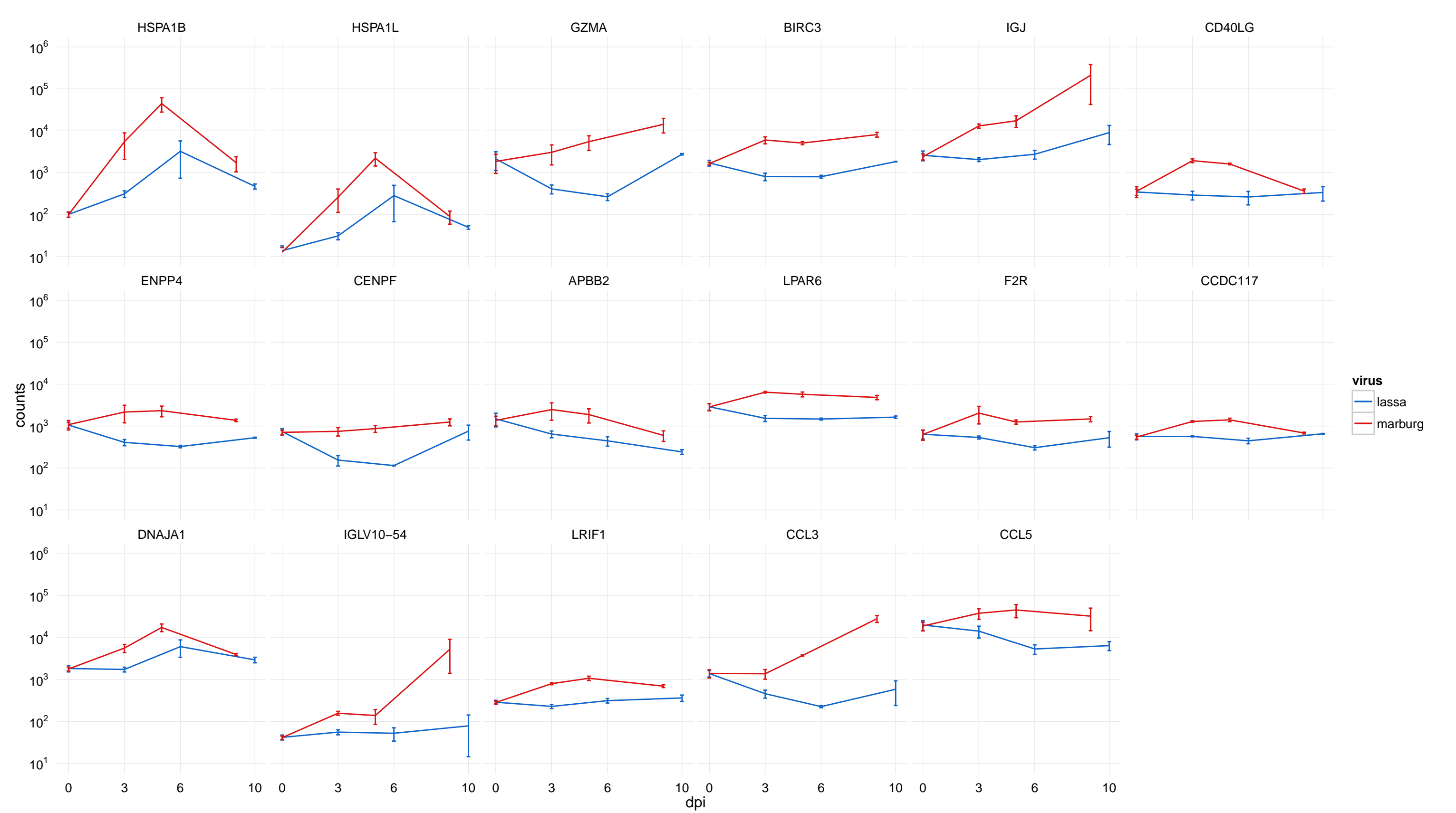

Supplement: Supplementary file 3 — Additional file 3: Visual representation of genes categorized as more highly expressed in Marburg. The x-axis represents time and the y-axis represents normalized read counts. (PDF 11 KB) [file 12864_2014_6648_MOESM3_ESM.pdf]
